# A novel high-dimensional NMR experiment for resolving protein backbone dihedral angle ambiguities

**DOI:** 10.1007/s10858-020-00308-y

**Published:** 2020-04-01

**Authors:** Clemens Kauffmann, Krzysztof Kazimierczuk, Thomas C. Schwarz, Robert Konrat, Anna Zawadzka-Kazimierczuk

**Affiliations:** 1 Max Perutz Laboratories, Department of Structural and Computational Biology, University of Vienna, Vienna Biocenter Campus 5, 1030 Vienna, Austria; 2 Faculty of Chemistry, Biological and Chemical Research Centre, University of Warsaw, Żwirki i Wigury 101, 02-089 Warsaw, Poland; 3 Centre of New Technologies, University of Warsaw, Banacha 2C, 02-097 Warsaw, Poland

**Keywords:** Cross-correlated relaxation, Dihedral angles, Chemical shift anisotropy, Dipolar interactions, Intrinsically disordered proteins, High-dimensional NMR experiments, Non-uniform sampling

## Abstract

Intrinsically disordered proteins (IDPs) are challenging established structural biology perception and urge a reassessment of the conventional understanding of the subtle interplay between protein structure and dynamics. Due to their importance in eukaryotic life and central role in protein interaction networks, IDP research is a fascinating and highly relevant research area in which NMR spectroscopy is destined to be a key player. The flexible nature of IDPs, as a result of the sampling of a vast conformational space, however, poses a tremendous scientific challenge, both technically and theoretically. Pronounced signal averaging results in narrow signal dispersion and requires higher dimensionality NMR techniques. Moreover, a fundamental problem in the structural characterization of IDPs is the definition of the conformational ensemble sampled by the polypeptide chain in solution, where often the interpretation relies on the concept of ‘residual structure’ or ‘conformational preference’. An important source of structural information is information-rich NMR experiments that probe protein backbone dihedral angles in a unique manner. Cross-correlated relaxation experiments have proven to fulfil this task as they provide unique information about protein backbones, particularly in IDPs. Here we present a novel cross-correlation experiment that utilizes non-uniform sampling detection schemes to resolve protein backbone dihedral ambiguities in IDPs. The sensitivity of this novel technique is illustrated with an application to the prototypical IDP $\alpha$-Synculein for which unexpected deviations from random-coil-like behaviour could be observed.

## Introduction

Cross-correlated relaxation (CCR) experiments have long been established as a unique tool to study protein structure and dynamics (Brutscher 2000; Kumar et al. 2000). CCR experiments quantify correlated interferences of dipolar (dd) and/or chemical shift anisotropy (CSA) interactions. The possibility to use these effects to study protein backbone geometry was first demonstrated in 1997 (Reif et al. 1997) by investigating the backbone angle $\psi$ from the dipole–dipole (dd–dd) interferences of ${\text{H}}^{\alpha}_{i}{\text{C}}^{\alpha} _{i}$– ${\text {H}^{\text{N}}_{i}}{\text {N}_{i}}$. Other CCR rates probing dihedral angles along the protein backbone were soon proposed, combining different dd–dd (Yang et al. 1998; Chiarparin et al. 1999; Pelupessy et al. 1999a, b; Chiarparin et al. 2000; Crowley et al. 2000; Pelupessy et al. 2003), dd-CSA (Yang et al. 1997, 1998; Chiarparin et al. 1999; Kloiber and Konrat 2000a, b) and CSA-CSA (Skrynnikov et al. 2000; Pelupessy et al. 2003) interactions, see (Schwalbe et al. 2001; Vögeli and Vugmeyster 2019) for more extensive overviews.

Every such combination of backbone interactions comes with its unique angular dependency, ranging from simple Karplus relations to more complex expressions. Due to its non-bijective nature, an isolated CCR rate does not allow to determine the underlying angle(s) unambiguously. However, by combining and analyzing multiple CCR experiments at once, these ambiguities were shown to be resolvable (Kloiber et al. 2002) if a singular average structure can be assumed. Intrinsically disordered proteins (IDPs) prove to be more challenging in this regard. Their conformational flexibility leads to highly averaged and thus ambiguous observables, i.e. the underlying structural ensemble is underdetermined. This poses major challenges: Conventional point estimate approaches are ill-equipped for modelling heterogeneous ensembles. Not only does the high dimensionality of the problem lead to a steep increase of computational effort but also the experimental underdetermination bears the inherent risk of overfitting. While both difficulties appear to be manageable by Bayesian and Maximum Entropy methods, implementation details and subtleties are still being investigated and improved (Mantsyzov et al. 2014, 2015; Olsson et al. 2013; Bonomi et al. 2016; Hummer and Köfinger 2015; Köfinger et al. 2019; Cesari et al. 2016, 2018; Rangan et al. 2018).

The second challenge lies in the limited number of observables. Despite the conceptual and technical advances made, the underdetermination of structural ensembles still can only be amended by combining an increasing amount of experiments. The above-mentioned studies resorted to well-established NMR experiments such as scalar couplings, chemical shifts, RDCs and/or NOEs.

We argue that CCR rates provide a unique and valuable source of information that has been largely overlooked in the past, with few exceptions (Stanek et al. 2013), mostly due to the experimental challenges involved. The chemical shifts of IDPs are distributed over a very narrow spectral range, reflecting the high mobility of the polypeptide chain; a nucleus experiences (at the fast time-scale) various chemical environments which leads to observation of an averaged chemical shift. Therefore, to achieve peak separation, high-dimensional experiments need to be employed. The above-mentioned CCR experiments were designed as two-dimensional (2D) or three-dimensional (3D), which do not necessarily provide sufficient resolution for IDPs. Taking into account that CCR measurements require quantitative analysis of peak intensities, which can be disturbed by even slight peak overlap, higher dimensionality is indispensable, as demonstrated by the four-dimensional (4D) C${^\prime }_{i}$ CSA—${\text {H}^{\text{N}}_{i}}{\text {N}_{i}}$dd CCR experiment of Stanek et al. (2013).

However, not only preexisting CCR experiments are worthy of consideration, the complex conformational averaging of IDPs demands the development of novel orthogonal experiments that complement existing ones. Surprisingly, most reported CCR experiments neglect the potential of dipolar interactions between not-covalently bound nuclei, Crowley et al. (2000) being the only exception. Thus, we aim to extend the palette of existing CCR experiments by an entirely new interaction: Interfering ${\text {H}^{\text{N}}_{i}}{\text {H}^{\alpha }_{i-1}}$dd and C${^\prime }_{i-1}$ CSA relaxation reveals a highly informative $\psi$-dependent observable with reduced ambiguity compared to more commonly used CCR rates and scalar couplings.

We verify the technique with Ubiquitin and demonstrate convincing agreement between structural parameters derived from PDB structures and experimental cross-correlation rates. As a first application to intrinsically disordered proteins, we show that the prototypical IDP $\alpha$-Synuclein displays surprising deviations from random-coil-like behaviour which is undetectable by conventional chemical-shift-based methods.

## Methods

### NMR experiment

**Fig. 1:**
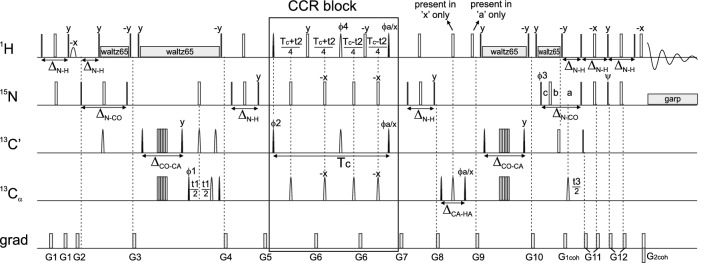
Pulse sequence of the experiment for the ${\text {H}^{\text{N}}_{i}}{\text {H}^{\alpha }_{i-1}}$dd – C${^\prime }_{i-1}$ CSA CCR rate measurement. $\text {C}^\alpha$ evolution is in real-time mode, C${^\prime }$ evolution is in constant-time mode and N evolution is in semi-constant time mode: $\text {a}=(\text {t}3+\varDelta _{\text{N-CO}})/2$, $\text {b}=\text {t}3(1-\varDelta _{\text{N-CO}}/\text {t}3^{\text{max}})/2$, $\text {c}=\varDelta _{\text{N-CO}}(1-\text {t}3/\text {t}3^{\text{max}})$. The delays were set as follows: $\varDelta _{\text{N-H}} = 5.4 \text { ms}$, $\varDelta _{\text{N-CO}} = 28 \text { ms}$, $\varDelta _{\text{CO-CA}} = 9.1 \text { ms}$, $\varDelta _{\text{CA-HA}} = 3.2 \text { ms}$, $\text {Tc} = 28 \text { ms}$. Unless noted explicitly, pulse phases are set to x. Phase $\phi a/x$ depends on the version of experiment; for reference experiment x, for cross experiment y. 16-step phase cycle was used: $\phi 1$ = x, -x, $\phi 2$ = 2(x), 2(-x), $\phi 4$ = 4(x), 4(y), 4(-x), 4(-y). Receiver phase $\phi rec=\phi 1 + \phi 2 + 2\cdot \phi 4$. Selective pulses affecting only $\text {H}^{\text{N}}$ nuclei were pc9 (Kupce and Freeman 1994) for $\pi /2$ pulses and reburp (Geen and Freeman 1991) for $\pi$ pulses. Simultaneous inversion of $\text {C}^\alpha$ and C${^\prime }$ spins was achieved using 6-element composite pulse (Shaka 1985). Selective pulses for C${^\prime }$ and $\text {C}^\alpha$ nuclei were q5 (for ${\pi/2}$ pulses) and q3 (for $\pi$ pulses) (Emsley 1969). The pulses labelled ”present in ’a’/’x’ only” were executed only in reference/cross version of the experiment, respectively

**Fig. 2:**
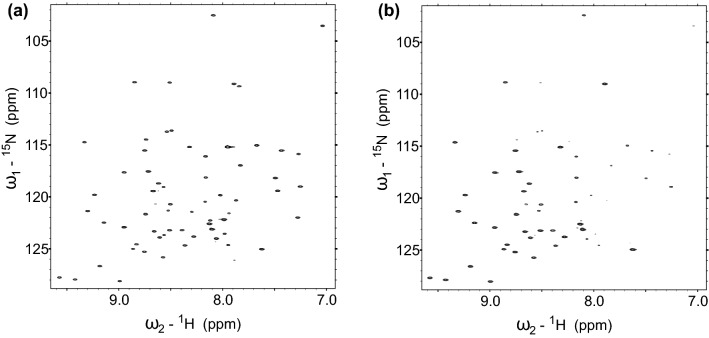
Comparison of 2D versions of the experiment (with no $\text {C}^\alpha$ and C${^\prime }$ evolutions) for the *reference* (**a**) and *cross* (**b**) experiments, showing differences in relative peak intensities of some residues

The pulse sequence of the experiment for ${\text {H}^{\text{N}}_{i}}{\text {H}^{\alpha }_{i-1}}$dd – C${^\prime }_{i-1}$ CSA CCR rate measurement is shown in Fig. 1. It includes three indirectly-detected dimensions ($\text {C}^\alpha _{i-1}$, C${^\prime }_{i-1}$ and $\text {N}_i$) and one directly-detected $\text {H}^{\text{N}}_{i}$ dimension. The employed method of CCR rate quantification is called quantitative spectroscopy, which means that two independent data sets (later referred to as *reference* and *cross*) are acquired. The coherence entering the CCR block of the pulse sequence is partially preserved and partially converted to another one. The conversion is CCR-mediated, thus the measurement of the intensities of signals originating from both components allows for quantification of the CCR effect. In the *cross* spectrum the observable magnetization originates from the converted coherence, while in the *reference* spectrum it originates from the preserved coherence. Crucial is the fact that the peak intensities in the *reference* spectrum are proportional to $\cosh {(\varGamma T_c)}$ where $\varGamma$ is the corresponding CCR rate and $T_c$ is the time of the CCR evolution, while in the *cross* spectrum they are proportional to $\sinh {(\varGamma T_c)}$ which is why their values ($I_{ref}$ and $I_{cross}$) are not identical (see Fig. 2). Therefore the CCR rates are calculated (separately for each residue of the protein under investigation) using the following formula:

1 $$\begin{aligned} \varGamma =\frac{1}{T_c} {{\,\text{arctanh}\,}}\left( \frac{I_{cross}}{I_{ref}} \right) \end{aligned}$$

Importantly, the formula should be modified if the *reference* and *cross* experiments were acquired with different number of scans, which is a good practice due to significantly lower sensitivity of the *cross* experiment. In such a case, the intensity ratio should be divided by the ratio of number of scans acquired in *cross* and *reference* experiments.

The coherence transfer pathway is shown below. The beginnings of *reference* and *cross* experiments are identical, up to the CCR block:

$$\begin{aligned}&{\text {H}^{\text{N}}_{i} \,{}_{\text{z }}}\rightarrow 2{\text {H}^{\text{N}}_{i} \,{}_{\text{z }}}\text {N}_i \,{}_{\text{z }}\rightarrow 2\text {N}_i \,{}_{\text{z }}\text {C}{^\prime}_{i-1} \,{}_{\text{z }}\\&\quad \rightarrow 4\text {N}_i \,{}_{\text{z }}\text {C}{^\prime}_{i-1} \,{}_{\text{z }}\text {C}^{\alpha }_{i-1} \,{}_{\text{z }}\xrightarrow []{C^{\alpha }_{i-1} evolution} 4\text {N}_i \,{}_{\text{z }}\text {C}{^\prime}_{i-1} \,{}_{\text{z }}\text {C}^{\alpha }_{i-1} \,{}_{\text{z }}\\&\quad \rightarrow 8{\text {H}^{\text{N}}_{i} \,{}_{\text{z }}}\text {N}_i \,{}_{\text{z }}\text {C}{^\prime}_{i-1} \,{}_{\text{z }}\text {C}^{\alpha }_{i-1} \,{}_{\text{z }}\end{aligned}$$

Then, during the CCR block, the product operator 8$${\text {H}^{\text{N}}_{i} \,{}_{\text{z }}}$$$$\text {N}_i \,{}_{\text{z }}$$$$\text {C}{^\prime}_{i-1} \,{}_{\text{z }}$$$$\text {C}^{\alpha }_{i-1} \,{}_{\text{z }}$$is partially preserved and partially converted into 16$${\text {H}^{\text{N}}_{i} \,{}_{\text{z }}}$$$$\text {N}_i \,{}_{\text{z }}$$$$\text {C}{^\prime}_{i-1} \,{}_{\text{z }}$$$$\text {C}^{\alpha }_{i-1} \,{}_{\text{z }}$$$$\text {H}^{\alpha }_{i-1} \,{}_{\text{z }}$$. This conversion occurs for total time $$T_c$$. Also, the evolution of $$\text {C}{^\prime }_{i-1}$$ nuclei occurs here. Importantly, coherence of no other CCR rate occurs during the CCR block, thus providing a clean result. After the CCR block, the coherence (8$${\text {H}^{\text{N}}_{i} \,{}_{\text{z }}}$$$$\text {N}_i \,{}_{\text{z }}$$$$\text {C}{^\prime}_{i-1} \,{}_{\text{z }}$$$$\text {C}^{\alpha }_{i-1} \,{}_{\text{z }}$$for the *reference* experiment and 16$${\text {H}^{\text{N}}_{i} \,{}_{\text{z }}}$$$$\text {N}_i \,{}_{\text{z }}$$$$\text {C}{^\prime}_{i-1} \,{}_{\text{z }}$$$$\text {C}^{\alpha }_{i-1} \,{}_{\text{z }}$$$$\text {H}^{\alpha }_{i-1} \,{}_{\text{z }}$$for the *cross* experiment) is gradually back-converted into an observable $$\text {H}^{\text{N}}_{i}$$ transverse magnetization. In particular, in the second INEPT after the CCR block, 8$$\text {N}_i \,{}_{\text{z }}$$$$\text {C}{^\prime}_{i-1} \,{}_{\text{z }}$$$$\text {C}^{\alpha }_{i-1} \,{}_{\text{z }}$$$$\text {H}^{\alpha }_{i-1} \,{}_{\text{z }}$$operator in *cross* version is converted into 4$$\text {N}_i \,{}_{\text{z }}$$$$\text {C}{^\prime}_{i-1} \,{}_{\text{z }}$$$$\text {C}^{\alpha }_{i-1} \,{}_{\text{z }}$$one using the $$J_{CA{-}HA}$$ scalar coupling. Notably, the operator involving a glycine residue contains two alpha protons and thus will not be converted into an observable magnetization using this pulse scheme. This is the reason why the presented experiment does not allow to determine CCR rates for glycine residues. The last indirect evolution (of $$\text {N}_{i}$$ nuclei) occurs during the 2$$\text {N}_i \,{}_{\text{z }}$$$$\text {C}{^\prime}_{i-1} \,{}_{\text{z }}$$$$\rightarrow$$ 2$${\text {H}^{\text{N}}_{i} \,{}_{\text{z }}}$$$$\text {N}_i \,{}_{\text{z }}$$INEPT block.

The proposed pulse sequence was designed in a way which precludes the evolution of any other CCR rate. Nonetheless, the results still may be perturbed by another factor, namely the dispersion of the scalar coupling constants between alpha carbon and alpha proton $J_{CA{-}HA}$ throughout the protein. After the CCR block, in the *cross* version of the experiment this coupling is evolved. It is however not evolved in the *reference* experiment. Therefore deviation from this assumed coupling constant will cause perturbation of the obtained $\varGamma$ value. For deviations of ± 5 % of the 146 Hz value of the J-coupling, the $\varGamma$ perturbation is not big and typically does not exceed 1.5 %.

An important issue is parameters of amide-proton selective pulses in the CCR block of the pulse sequence. The excitation range, defined using ’offset’ and ’bandwidth’ parameters of the pulse, should cover the whole amide-proton region, but not overlap with the alpha-proton region. For residues with $H^N$ outside or $H^{\alpha }$ inside the excitation range, the measured ratio of peak intensities from reference and cross spectra will not provide a correct value of the CCR rate. As the resonance assignment is already known at the stage of CCR rates measurements, it is possible to adjust the offset and/or bandwidth to match the particular protein. In general, the problem is less pronounced for IDPs, which feature narrower chemical shift ranges, than for folded proteins. The values used in the experiments shown in the present study (offset of 8.3 ppm and bandwidth of 3.5 ppm) matched well both proteins used, Ubiquitin and $\alpha$-Synuclein. The $H^N$ of one of the Ubiquitin residues (Ile36) was outside of the excitation range, but the peak involving this nucleus provides information on the CCR rate of the preceding Gly35 residue, which—being a glycine—is not useable anyway.

The pulse sequence of the presented experiment can be obtained from the authors upon request.

### Data analysis

The expected angular dependence was modelled in accordance with Yang et al. (1997) assuming model-free dynamics (Lipari and Szabo 1982),

2 $$\begin{aligned} \varGamma ^{DD,CSA}_{AB,C}(\psi ,\theta (\psi )) = \frac{4}{15} \frac{\mu _0 \hbar }{4\pi } \frac{\gamma _A\gamma _B}{r^3_{AB}(\psi )} B_0 \gamma _{C} \times f_C \times \tau _c S^2, \end{aligned}$$

where

3 $$\begin{aligned} f_C= & {} \frac{1}{2}[\sigma _{xx}(3\cos ^2\theta _{AB,X}-1)+\sigma _{yy}(3\cos ^2\theta _{AB,Y}-1)\nonumber \\&+\sigma _{zz}(3\cos ^2\theta _{AB,Z}-1)], \end{aligned}$$

with $A=H^N_i$, $B=H^\alpha _{i-1}$ and $C = C{^\prime }_{i-1}$. $\gamma$ is the gyromagnetic ratio, $\mu _0$ is the vacuum permeability, $\hbar$ is the reduced Planck constant, $B_0$ is the magnetic field strength, $\tau _c$ is the global correlation time, $S^2$ is the local order parameter, $\sigma _{xx,yy,zz}$ are the tensor components of the diagonal CSA tensor (in ppm), $r_{AB}$ is the internuclear distance between *A* and *B*, $\theta$ denotes the projection angles between the dipolar unit vector *AB* and the principal axes *X*, *Y*, *Z* of the CSA tensor coordinate system.

$\varGamma$ as a function of $\psi$ was calculated numerically using an Avogadro-generated (Hanwell et al. 2012) backbone geometry with $\psi = -180^{\circ }$ (Table 1) and rotating around the $\text {C}^\alpha$–$\text {C}{^\prime }$ bond in $1^{\circ }$ steps.

**Table 1:** The model protein backbone in x,y,z-coordinates with $\psi = \omega = -180^{\circ }$

Atom	x (Å)	y (Å)	z (Å)
$C^{\alpha }_0$	0.00000	0.00000	0.00000
$H^{\alpha }_0$	$-$ 0.33381	0.81771	$-$ 0.46895
$C{^\prime }_0$	$-$ 0.52182	$-$ 0.01279	1.43821
$O_0$	0.26150	$-$ 0.02925	2.38639
$N_1$	$-$ 1.84661	$-$ 0.00472	1.55557
$H^N_1$	$-$ 2.47453	0.06788	0.78069

Parameters were adapted primarily from Engh and Huber (2006), angles involving hydrogens were taken from Momany et al. (1975). The principal axes of the carbonyl CSA tensor were set in accordance with Teng et al. (1992): The Z-axis was defined as the cross product of the $\text {C}{^\prime }$–$\text {O}$ and the $\text {C}{^\prime }$–$\text {C}^{\alpha }$ bond unit vectors, the X- and Y-axis as clockwise rotations of the $\text {C}{^\prime }$–$\text {O}$ bond unit vector around the Z-axis by 82$^{\circ }$ and − 8$^{\circ }$, approximating the $\text {O}$–$\text {C}{^\prime }$–$\text {N}$ angle with 120$^{\circ }$.

The tensor components of Ubiquitin were adapted from Cisnetti et al. (2004). $\sigma _{xx}$ and $\sigma _{zz}$ were set according to the reported averages as 249.4 ppm and 87.9 ppm. Using the suggested calibration, the average $\sigma _{yy}$ was derived from the chemical shifts (BMRB ID 17769, Cornilescu et al. (1998)) as 191.1 ppm. Uncertainties were estimated by allowing the tensor components to vary within their reported standard deviations (*x*, *y*, *z* = 6.1, 6.1, 5.4 ppm) while still matching the chemical shift range, yielding a lower limit of $\sigma _{xx},\sigma _{yy},\sigma _{zz}$ = 243.3, 211.7, 87.4 ppm and an upper limit of $\sigma _{xx},\sigma _{yy},\sigma _{zz}$ = 255.5, 172.9, 93.3 ppm.

A correlation time $\tau _c$ of 4.1 ns was assumed (Schneider et al. 1992), order parameters were taken from Tjandra et al. (1995) with an average $S^2$ of 0.84, a lower limit of 0.70 and an upper limit of 0.91 (excluding the reported outlier of 0.565 at L73).

Neighbour-corrected Structural Propensity Calulator (ncSPC) values for Ubiquitin and $\alpha$-Synuclein (BMRB ID 17769 and 6968, respectively) were calculated using the tool of Tamiola and Mulder (2012) with default settings and the Tamiola et al. (2010) library.

CCR rates expected for random-coil-like residues were estimated using the random coil library of Mantsyzov et al. (2015) with a total of 152870 $\psi$-angles (excluding glycine and proline residues). Rates were calculated according to Equation 2 and averaged. The effective correlation time was estimated from the experimentally observed values: The range of Ubiquitin (0.67 to 10.39 $\text {s}^{-1}$) was normalized to the mean correlation time ($3.44 \text { ns} = \tau _c S^2$). Dividing the observed range of $\alpha$-Synculein (1.38 to 8.16 $\text {s}^{-1}$) by this factor yields an estimate for the effective correlation time of 2.40 ns. Thus, the effective correlation time was estimated to lie within 2 and 3 ns, equating to an expected $\varGamma$ for random-coil-like residues between 3.10 and 4.66 $\text {s}^{-1}$.

## Experimental

The sample of 1 mM ${}^{13}\text {C}$,${}^{15}\text {N}$-uniformly labeled Ubiquitin dissolved in 10 mM potassium phosphate buffer of pH$= 6.5$ was purchased from ASLA Biotech. The sample of 1.35 mM ${}^{13}\text {C}$,${}^{15}\text {N}$-uniformly labeled $\alpha$-Synculein was produced using the protocol of Wrasidlo et al. (2016). The protein was equilibrated in 20mM sodium phosphate buffer at pH 6.5 containing 200mM NaCl, 0.5mM EDTA, 0.02% $\text {NaN}_{3}$ and 1x cOmplete^TM^ Protease Inhibitor Cocktail (Roche) with addition of 10% $\text {D}_{2}\text {O}$ for lock.

All the experiments were performed on a Bruker AVANCE III HD 800 MHz spectrometer equipped with a 5 mm TCI-HCN cryo-probe. The experiments for Ubiquitin were performed at 298 K and for $\alpha$-Synculein—at 284.5 K. The experimental parameters are gathered in Table 2.

The amide-proton selective pulses employed in the CCR block of the pulse sequence were defined within the pulse program using WaveMaker. Offset was set equal to 8.3 ppm and bandwidth to 3.5 ppm.

Data from conventional experiments were processed using the fast Fourier transform algorithm implemented in mddnmr software (Orekhov et al. 2004–2019). Data from NUS experiments were processed with compressed sensing (Kazimierczuk and Orekhov 2011), using the iterative soft thresholding algorithm implemented in mddnmr (Orekhov et al. 2004–2019) with parameters set to obtain good quantitativeness, i.e. 500 iterations and parameter $\lambda = 0.01$ (resulting in a relative threshold change from 1.00 in the first iteration to 0.995 in the final one). The data were displayed and analyzed using Sparky (Goddard and Kneller 2002).

**Table 2:** Experimental parameters for all data sets (dim—dimensionality, ni—number of hypercomplex increments, ns—number of scans, conv—conventional sampling scheme, NUS—non-uniform sampling scheme)

Sample	dim	ni	$\text {C}^\alpha$		C${^\prime }$		N		Version	ns	Time, h
			sw,Hz	$t_{max}$,s	sw, Hz	$t_{max}$,s	sw,Hz	$t_{max}$,s			
Ubiq.	2D	335 (conv)	–	–	–	–	2550	0.1314	ref	4	1 h 10 min
									cross	80	23 h
Ubiq.	3D	3536 (conv)	–	–	3000	0.0173	2550	0.0267	ref	4	23 h 20 min
									cross	8	46 h 45 min
Ubiq.	4D	680 (NUS)	6000	0.01	3000	0.0173	2550	0.0267	ref	4	9 h
									cross	28	63 h
$\alpha$-syn.	4D	600 (NUS)	5600	0.0098	2200	0.0172	2700	0.0278	ref	4	8 h
									cross	28	55 h 45 min

## Results and discussion

**Fig. 3:**
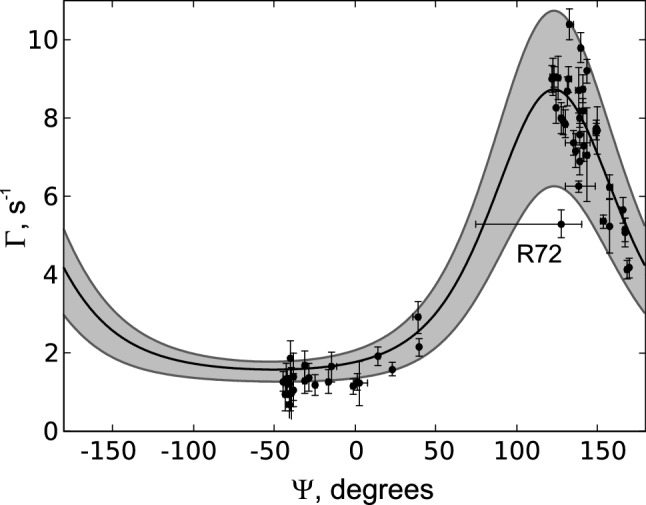
Comparison of the measured and expected CCR rates $\varGamma _{{\text {H}^{\text{N}}_{i}}{\text {H}^{\alpha }_{i-1}},C{^\prime }_{i-1}}$ of Ubiquitin, disregarding glycine residues. Measured rates are plotted as black dots against their reported average angle $\psi$ (PDB code 1d3z). The expected average rate with tensor components *xx*, *yy*, *zz* = 249.4, 191.1, 87.9ppm, $\tau _c$ = 4.1 ns and $S^2$ = 0.84, is represented by the black line. Expected variations are depicted in grey, with a lower limit of *xx*, *yy*, *zz* = 243.3, 211.7, 87.4ppm and $S^2$ = 0.70 and an upper limit of *xx*, *yy*, *zz* = 255.5, 172.9, 93.3ppm and $S^2$ = 0.91. Horizontal error bars indicate the reported range of angles, vertical error bars are estimated from spectral noise

In order to establish the method as a reliable tool for $\psi$ angle determination, the experiment was first tested on a protein of known structure, Ubiquitin. Using a 3D version of the experiment (no $\text {C}^\alpha _{i-1}$ evolution) with conventional sampling of the evolution time space, the consistency of the obtained $\varGamma$ rates with the solution structures (10 in total) of Ubiquitin (Cornilescu et al. 1998), PDB code 1d3z, was checked. In Fig. 3 the experimentally obtained $\varGamma$ rates (for each residue) are plotted against the average $\psi$ angles of the protein structures, horizontal bars indicate the reported range of $\psi$ values. The experimental uncertainties, shown on the plot as vertical error bars, were estimated based on the spectral noise, calculated in Sparky as a median of absolute values of 10000 randomly chosen spectral points, using the uncertainty propagation method. The potential errors originating from variation of the $J_{CA{-}HA}$ scalar coupling values throughout the protein are not shown. Assuming $J_{CA{-}HA}$ deviated by 5 % from 146 Hz, the resulting uncertainty would be around 1/5 of the noise-originating uncertainties. The theoretically expected $\psi$ dependence is shown in black, the grey area depicts the uncertainty estimate due to variations of the CSA tensor and the local order parameter (see “Methods” section). Note that the rates could not be quantified for all residues: Besides glycines (see “Methods” section), CCR rates could not be determined for three residues preceding prolines as well as seven other residues (Thr7, Leu8, Ile23, Phe45, Asp52, Leu73, Arg74) due to a very low peak intensity in the reference spectrum.

Clearly, the measured rates agree well with the rates calculated from the PDB structures. The apparent outlier of the highlighted residue R72 is to be expected. As part of the flexible C-terminal tail, its dynamics and structural averaging are reflected both in a low $\varGamma$ and the horizontal uncertainty range reported in the PDB structures.

**Fig. 4:**
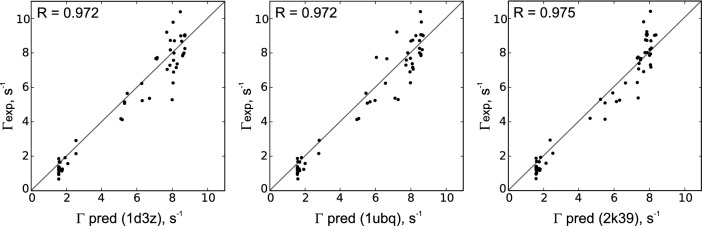
Comparison of calculated CCR rates of three different PDB structures of Ubiquitin (x axes) with the experimentally obtained CCR rates (y axes). R is the Pearson correlation coefficient

To further assess the influence of $\psi$ uncertainties, a comparison of different PDB entries is shown in Fig. 4: The aforementioned set of NMR derived solution structures 1d3z, the crystal structure 1ubq (Vijay-Kumar et al. 1987) and the ensemble 2k39 (Lange et al. 2008). Average CCR rates were calculated for all structures and compared to the experimentally obtained rates. While the overall agreement between them is apparent, it is worth noting that the ensemble of Lange et al. (2008) (116 structures) gives the highest Pearson R of 0.975. We conclude that the observed deviations are mostly due to the aforementioned experimental and theoretical uncertainties as indicated in Fig. 3.

As Fig. 3 illustrates, the $\varGamma$ rate can be consistent with two different $\psi$ angles. To resolve these ambiguities and determine the correct conformation, additional experiments need to be analyzed in parallel. Still, a qualitative interpretation is straightforward: Low rates correspond to helical motifs, high rates to $\beta$-strands. Note that the low range between ca. − 140$^\circ$ and 50$^\circ$ is less steep and pronounced. Thus, $\psi$ angles corresponding to $\beta$-like elements can be determined with higher precision than angles of helical residues.

**Fig. 5:**
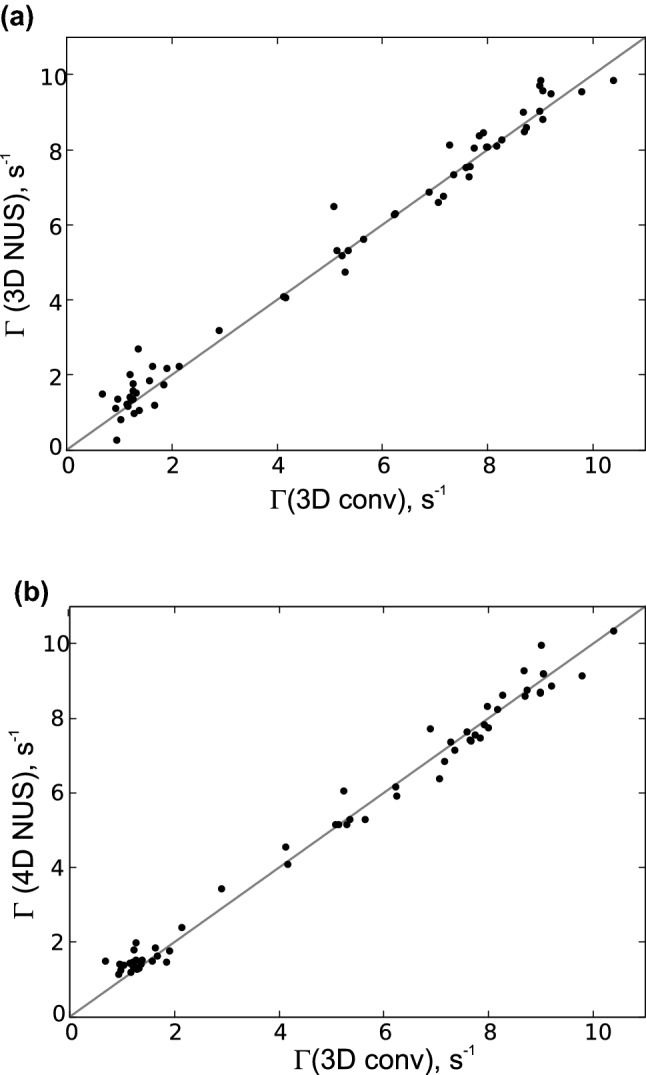
Comparison of CCR rates values for Ubiquitin sample obtained using 3D conventional (fully-sampled) data with the values obtained using: **a** a subset of 680 random points chosen from the 3D conventional data set, **b** 4D NUS dataset

Recording the experiment in its 4D version, suitable to crowded spectra of IDPs, requires using of non-uniform sampling (NUS). We have chosen the compressed-sensing IST algorithm for NUS data processing. To check the reliability of the CCR rates calculated using spectra reconstructed from NUS data, we compared the CCR rates of Ubiquitin from two sparse data sets with the rates obtained from the 3D conventional spectra. One sparse data set was obtained by randomly choosing a subset of points from a conventional 3D data set, while another was a 4D dataset recorded in a NUS manner. The comparison proving the methods reliability is shown in Fig. 5.

**Fig. 6:**
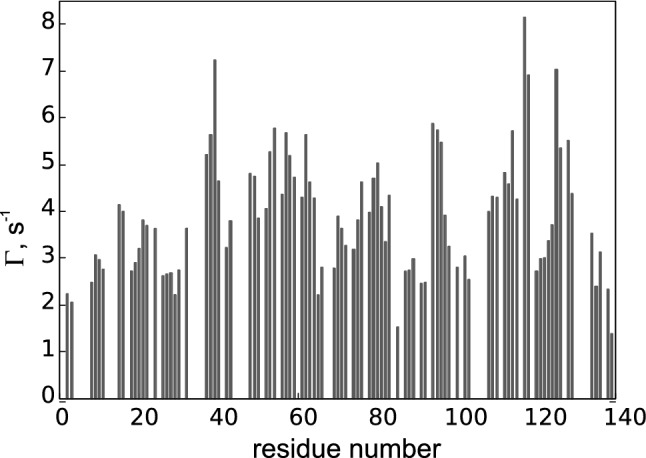
$\varGamma _{{\text {H}^{\text{N}}_{i}}{\text {H}^{\alpha }_{i-1}},C{^\prime }_{i-1}}$ CCR rates of $\alpha$-Synculein obtained using the 4D version of the proposed experiment

Finally, the experiment was acquired for the model IDP $\alpha$-Synculein as a prototypical showcase. CCR rates could be determined for 92 residues (see Fig. 6), which constitutes 80 % of non-glycine residues for which the $C{^\prime }_{i-1}$–$N_{i}$–$H^N_{i}$ chemical shifts were known from the BMRB entry 6968. The remaining 20 % of peaks (i.e. 23 peaks) were overlapped. Importantly, this still represents a noticeable decrease compared to the 3D $H^N$–*N*–$C{^\prime }$ projection in which a total of 30 residues are overlapping.

**Fig. 7:**
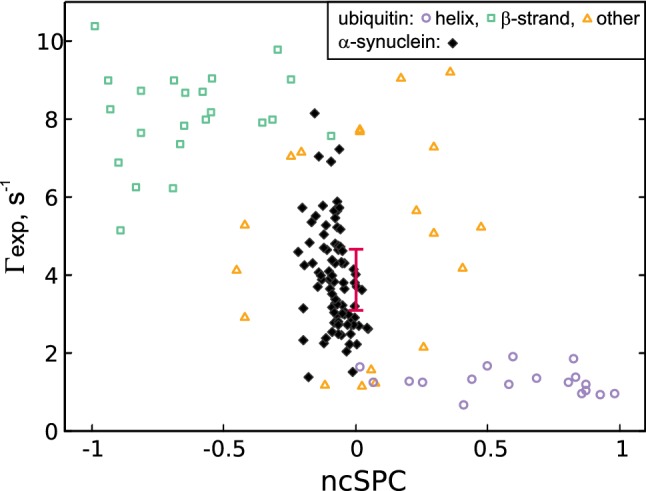
$\varGamma _{{\text {H}^{\text{N}}_{i}}{\text {H}^{\alpha }_{i-1}},C{^\prime }_{i-1}}$ rates of Ubiquitin and $\alpha$-Synuclein plotted against their corresponding ncSPC scores. The data corresponding to Ubiquitin residues is plotted with empty markers corresponding to their secondary structure (as in PDB entry 1d3z): helix—circle, $\beta$-strand—square, other—triangle. The data for $\alpha$-Synculein is plotted using filled markers. Expected values for random-coil-like residues are indicated by the error bar: Centered at 0 ncSPC, the bar indicates the expected range for $\varGamma$ (as described in the Methods—“Data analysis” section)

It should be noted that due to conformational averaging these rates cannot be related to a single dihedral angle $\psi$ as in Fig. 3, however, the same underlying functional form is applicable. To assess the potential structural information content of the ${\text {H}^{\text{N}}_{i}}{\text {H}^{\alpha }_{i-1}}$dd—$\text {C}{^\prime }_{i-1}$ CSA CCR rate, we use the chemical shift derived structural propensity score provided by the neighbour-corrected Structural Propensity Calculator (ncSPC) (Tamiola and Mulder 2012) for both Ubiquitin and $\alpha$-Synuclein, see Fig. 7. Referring to the globular protein Ubiquitin first, we can see that both ncSPC and $\varGamma _{{\text {H}^{\text{N}}_{i}}{\text {H}^{\alpha }_{i-1}},C{^\prime }_{i-1}}$ allow to distinguish between helical and $\beta$-strand structures. For helical residues, the ncSPC values are positive and the corresponding CCR rates are below 2 $\text {s}^{-1}$. In contrast, for $\beta$-strand residues ncSPCs are negative and CCR rates exceed 5 $\text {s}^{-1}$. The observed vertical spread for residues located in the $\beta$ region is much higher than in helices which is expected given the underlying functional form of $\varGamma$ (Fig. 3). Interestingly, secondary structure motifs other than helix and strand show a noticeable vertical and horizontal spread, illustrating the shortcomings of chemical shift derived secondary structure assessments. Nevertheless, the observed increase in resolution is worth noting and indicates an interesting (and potentially valuable) complementarity between CCR rates and ncSPCs for elucidating conformational averaging in mobile parts of (folded) proteins.

The IDP $\alpha$-Synculein shows a strikingly different pattern. As expected, the ncSPC range is substantially narrower (− 0.22 up to 0.05) compared to a globular protein (due to structural averaging), which shows that no secondary structure motifs are constantly adapted. However, the CCR rates are significantly more diverse, ranging from 1.38 to 8.16 $\text {s}^{-1}$, thus exceeding our estimates for random-coil-like residues of 3.10 to 4.66 $\text {s}^{-1}$). This surprising heterogeneity indicates that residues in $\alpha$-Synculein are far from identical, but rather showing differential local structural propensities which are not displayed by the chemical shifts. We thus conclude that the analysis of CCR rates offers unique possibilities to investigate structural dynamics of IDPs in solution.

## Conclusion

A novel pulse sequence was presented that allows quantifying cross-correlated relaxation between the ${\text {H}^{\text{N}}_{i}}{\text {H}^{\alpha }_{i-1}}$dipole–dipole and the $\text {C}{^\prime }_{i-1}$ chemical shift anisotropy. Validated against Ubiquitin, this $\psi$-dependent interference proves to be highly sensitive and capable to distinguish between helical and $\beta$-strand structural elements. This is due to its non-trivial angular distance dependency, which results in a strongly decreased ambiguity compared to other commonly used scalar couplings and CCR rates.

We argue that this unique feature is of particular importance in the context of intrinsically disordered proteins (IDPs), where quantitative analysis is challenging due to the effects of ensemble averaging. Using $\alpha$-Synuclein as an example, we show that the proposed CCR rate is not only highly specific to structural motifs of folded proteins but maintains its functional range even in highly disordered proteins. These findings suggest surprising deviations from the random-coil-like behaviour chemical-shift-based methods would suggest. This might not only be due to the unique geometrical dependency of the CCR rate but also due to its sensitivity to local dynamics. While chemical shifts report on simple population averages, relaxation rates are relatively weighted by their effective correlation time. Thus, the contribution of compact substates, even if sparsely populated, is amplified by their dynamics. We conclude that CCR rates are uniquely suited to characterize IDP ensembles and especially their compact substates. A systematic approach combining various CCR rates to characterize backbone dihedral angle distributions of IDPs as well as folded proteins is currently under investigation in our lab.
